# Supplementary material for: Opportunistic screening for atrial fibrillation by clinical pharmacists in UK general practice during the influenza vaccination season: A cross-sectional feasibility study
Source: PLoS Med. 2020 Jul 17;17(7):e1003197. doi: 10.1371/journal.pmed.1003197 (PMC7367445; doi:10.1371/journal.pmed.1003197)
Supplement: S1 Supporting Information — Probabilistic sensitivity analysis, Markov simulation model, Monte Carlo simulation model. (DOCX) [file pmed.1003197.s002.docx]

**Cost-effectiveness Evaluation**

The economic analysis of Pharmacists Detecting Atrial Fibrillation (PDAF) intervention employed a Markov simulation model built using the cost-utility template by Edlin *et al.* [1], the National Institute for Health and Care Excellence (NICE) costing report for atrial fibrillation (AF) [2], and the methodology adapted from two previous AF screening studies [3, 4]. The costs of screening and new diagnosis of AF per individual were estimated from the total population of individuals aged 65 and above in England and Wales (a population of 10,517,461) [5, 6] assuming the prevalence of previously undiagnosed possible AF identified by the cardiologist’s interpretation of single-lead electrocardiogram (_SL_ECG) of 1.3% and the screening participation rate of 50% at base case [3]. The costs were adjusted for UK inflation in 2019 and included the purchasing cost of Kardia Mobile® devices (KMD), clinical pharmacist time [7], relevant medical interventions (12-lead ECG/GP interpretation and GP/cardiologist appointments for new AF) [8, 9], the cost of oral anticoagulant (OAC) therapy, ischaemic strokes/major bleeds [2] and false positive (AF/unreadable/unclassified) diagnoses when using either KMD or pulse palpation. The model compared a hypothetical cohort of patients with AF aged ≥ 65 who underwent screening (the intervention group), had an opportunity to be identified as having AF and were offered anticoagulation, with a cohort of patients that did not undergo screening and were therefore not offered stroke prevention (the alternative scenario; S1A_Fig). The incremental cost-effectiveness ratios (ICERs) were defined as the mean incremental costs divided by the mean incremental quality-adjusted life years (QALYs) when comparing the intervention group and the alternative scenario [9] and were presented as a mean (95% confidence intervals). The intervention was considered to be cost-effective if the estimated ICER was under the willingness-to-pay threshold of £20,000/QALY proposed by NICE [10]. The incremental net benefit (INB) per patient with new AF identified through screening was calculated as the incremental QALYs multiplied by £20,000 minus the incremental costs [9]. The detailed breakdown of costs and model assumptions is provided below.

^
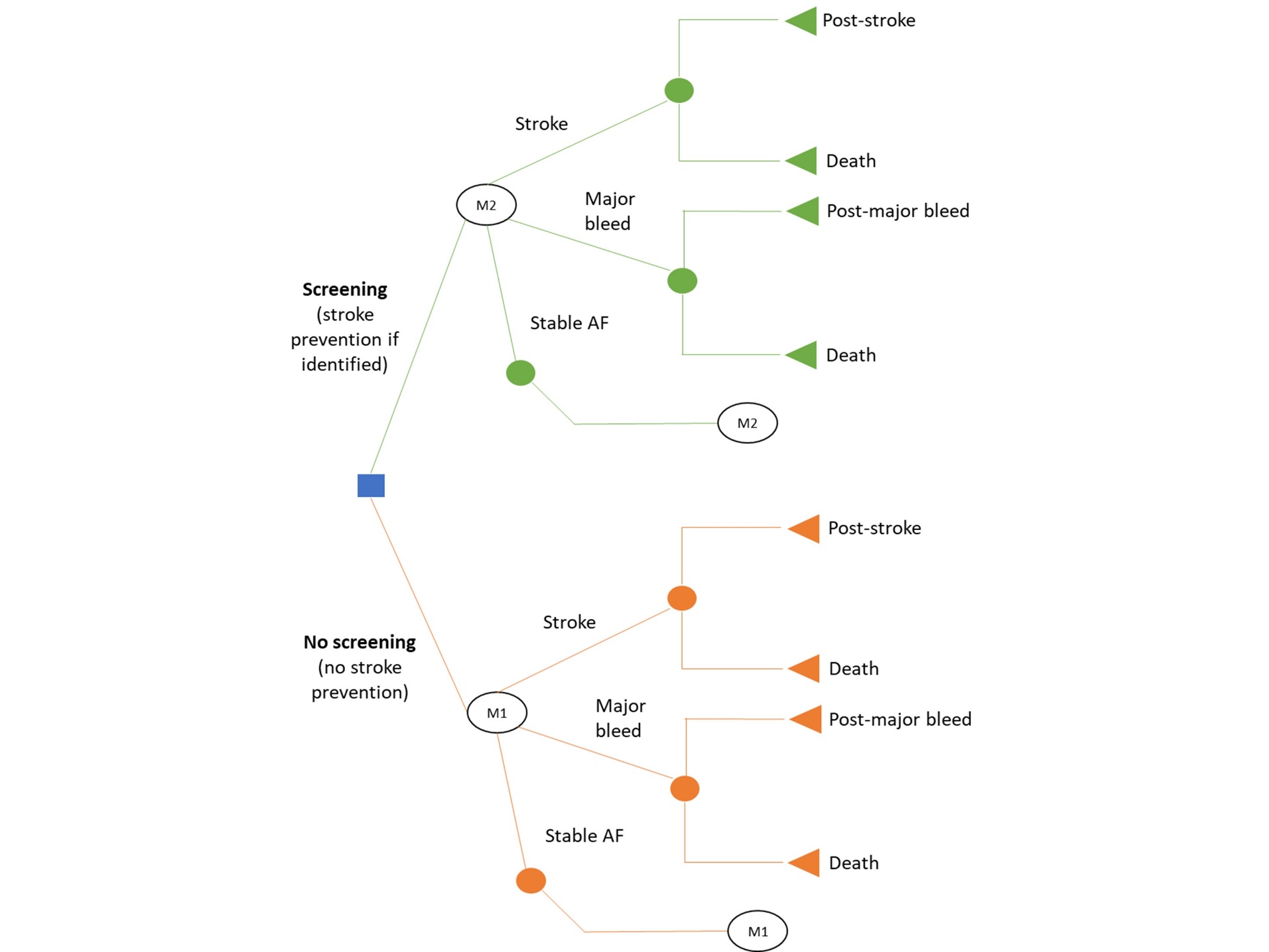
^

**S1A_Fig: Cost-effectiveness evaluation decision tree of PDAF study.**

M1 and M2 refer to Markov Model 1 and Markov Model 2, respectively. N.B. ‘stroke’ includes all incidences of ischaemic stroke.

**Results**

The base case ICER per QALY was £ (GBP) 14,460 [95% CI: £2,255 – £26,665] with KMD and £16,678 [95% CI: £7,191 – £26,164] with pulse palpation (S1A_Table). ICERs were improved with increasing adherence rates. The model was not sensitive to changes in the proportions of patients initiated on direct-acting oral anticoagulant (DOAC) and vitamin K (VKA) therapies, however was affected by the level of participation in the screening and by the percentage of unclassified/unreadable diagnoses. Where participation was maximised to 80% or the rate of unclassified/unreadable diagnoses was reduced by a half (to 6.7%), the base-case ICERs using KMD decreased to £8,902 [95% CI: -£9,661 – £27,465] and £12,286 [95% CI: -£1,169 – £25,741], respectively. At base case assumptions, the AF screening strategy proposed was found to be cost-effective in 71.8% and 64.3% of the estimates using KMD and pulse palpation, respectively. The INB compared to no screening strategy was £1,903/patient with AF and £120,084,946/all patients with new AF across England and Wales identified through screening by using KMD, and £946/patient with AF and £49,741,500/all patients with new AF identified by using pulse palpation (see S1B_Fig).

**S1A_Table: Summary of findings from the probabilistic sensitivity analysis (PSA) conducted as part of the cost-effectiveness evaluation of PDAF AF screening strategy.**

The table includes the key assumptions of the base case and presents the deviations from the base case tested during the PSA. It also displays the impact of the level of adherence to anticoagulant therapy on the cost-effectiveness of AF screening. The incremental cost-effectiveness ratios (ICERs) are expressed as a mean (95% confidence intervals).

| **Base Case Assumptions** | **Level of Adherence to Oral Anticoagulant Therapy (%)** | | | | |
| --- | --- | --- | --- | --- | --- |
|  | **40** | **55**  **(base case)** | **60** | **70** | **80** |
| - 3-monthly AF screening cost per participant with new diagnosis £286.96 - Total prevalence of AF 4.3% - Prevalence of previously undiagnosed AF 1.3% - Rate of Unclassified/Unreadable diagnoses 13.4% - Participation in screening rate 50% - Test sensitivity 92.3% - Test specificity 97.4% - %Patients on DOAC 56% - %Patients on VKA 44% | £19,957 (-£15,292-£55,207) | £14,460 (£2,255-£26,665) | £13,226 (-£1,288-£27,740) | £11,295 (£8,609-£13,981) | £9,824 (-£7,167-£26,815) |
| **Deviations from Base Case** | | | | | |
| - Pulse palpation instead of device - Rate of Unclassified/Unreadable diagnoses 2.2% - Test sensitivity 76.9% - Test specificity 92.2% | £23,030 (-£80,292-£126,351) | £16,678 (£7,191-£26,164) | £15,342 (£5,776-£24,907) | £13,046 (-£534-£26,627) | £11,356 (£5,794-£16,919) |
| - %Patients on DOAC 29% - %Patients on VKA 71% | £19,606 (£232-£38,981) | £14,127 (-£1,040-£29,293) | £12,956 (£5,634-£20,277) | £11,073 (-£32,704-£54,851) | £9,589 (£2,464-£16,713) |
| - Base-case assumptions - Screening participation rate 80% | £12,383 (£7,753-£17,012) | £8,902 (-£9,661-£27,465) | £8,164 (£85-£16,243) | £6,935 (£448-£13,421) | £6,026 (£366-£11,686) |
| - Base-case assumptions - Screening participation rate 30% | £33,494 (-£504-£67,493) | £24,300 (£6,982-£41,619) | £22,214 (£2,993-£41,434) | £19,609 (£10,450-£27,678) | £16,604 (£5,267-£27,941) |
| - Rate of Unclassified/Unreadable diagnoses 6.7% | £16,983 (£5,094-£28,872) | £12,286 (-£1,169-£25,741) | £11,225 (£3,091-£19,359) | £9,598 (-£7,832-£27,027) | £8,335 (-£9,500-£26,171) |


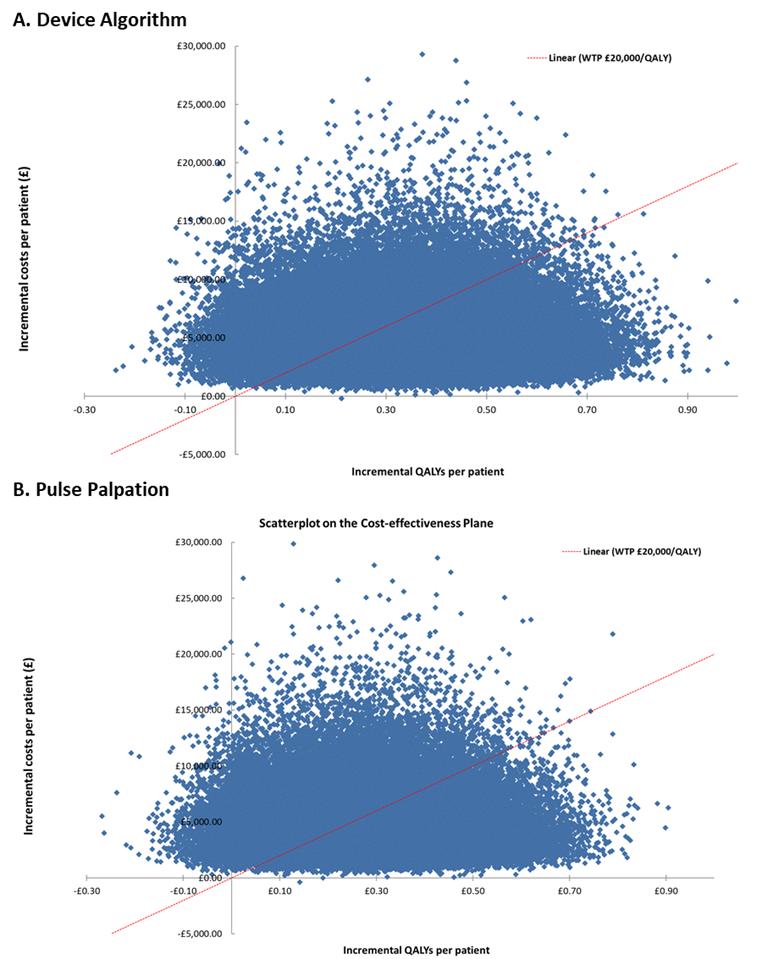


**S1B_Fig**: Incremental cost-effectiveness plane showing 100,000 pseudorandom Monte Carlo estimates of incremental costs and quality-adjusted life-years (QALY) gained per patient with AF when comparing: A. the base-case of the screening strategy using KMD with no screening; B. the base-case of the screening strategy using pulse palpation with no screening. Any points falling below the dotted line have an ICER < £20,000 per QALY gained. At base-case assumptions, AF screening strategies using the KMD and pulse palpation were found to be cost-effective in 71.8% and 64.3% of the estimates, respectively. The net benefit compared to no screening strategy was £1,903/patient with AF and £120,084,946/all patients with new AF across England and Wales over 10 years identified by using KMD, and £946/patient with AF and £49,741,500/all patients with new AF identified by using pulse palpation. Abbreviations: ECG – electrocardiogram; ICER – incremental cost-effectiveness ratio; KMD – Kardia Mobile® devices; WTP – willingness to pay [threshold].

**Detailed Breakdown of Costs and Model Assumptions**

The Markov simulation model utilised for this cost-effectiveness evaluation included a comparison of two hypothetical cohorts of individuals with atrial fibrillation (AF) aged 65 and over derived from the total population of England and Wales in 2016 (a population of 10,517,461) [5, 6]. One cohort of participants with AF (the intervention cohort) underwent screening and had an opportunity to be identified for oral anticoagulation therapy (OAC) whereas the other cohort (the alternative scenario) did not engage in AF screening and were therefore not identified for treatment. Patients with AF in the intervention cohort who were not identified through screening (false negatives) were included in the model and experienced the same risk of stroke or mortality as those who did not participate in the screening (the alternative scenario). The complete breakdown of costs and parameters used for the model is presented in S1B-S1D_Tables. The key assumptions of this model included:

- Participants with AF identified during the screening were assumed to display the same risk profile of ischaemic stroke and mortality as those with AF incidentally detected during routine care.
- The participation in screening rate was assumed to be 50% as in the cost-effectiveness study by Lowres and co-workers [3].
- Patients with AF who did not participate in the screening (the alternative scenario or 50% of the total model population) were assumed to not be identified through routine screening and displayed the risk of stroke and mortality observed in patients with AF who do not receive OAC.
- The total prevalence of AF was assumed to be 4.3% based on the cardiologist’s interpretation of single-lead ECG (_SL_ECG) recorded using Kardia Mobile® devices (KMD) during the Pharmacists Detecting Atrial Fibrillation (PDAF) study.
- The prevalence of ‘new’ AF was based on the diagnostic sensitivity of each index test and the prevalence of previously undiagnosed AF with reference to the cardiologist’s interpretation of _SL_ECG recorded using the KMD during the PDAF study (1.3%). Note that the median time between the screening using KMD and the follow-up confirmatory _12L_ECG was 16.0 [11.0; 24.0] days, and at the time of _12L_ECG, the prevalence of ‘new’ AF was 0.7%. The former value of 1.3% was used for cost-effectiveness analysis to ensure that all participants who may have presented with paroxysmal AF at the time of screening were considered in accordance with the European Society of Cardiology guidance, which indicates that a 30-second ECG recording of AF is diagnostic [11].
- The rate of Unclassified/Unreadable diagnoses was assumed to be 13.4% as determined by the _SL_ECG interpretation using the KMD algorithm during the PDAF study. The cost of these diagnoses was added to the cost of false positive AF diagnoses determined by the specificity of the KMD device compared to the study cardiologist.
- The sensitivity of KMD test for the detection of AF was assumed to be 92.3% using the cardiologist’s interpretation of _SL_ECG during the PDAF study as a reference standard.
- The specificity of KMD test for ruling out AF was assumed to be 97.4% using the cardiologist’s interpretation of _SL_ECG during the PDAF study as a reference standard.
- All participants with AF were assumed to be eligible for OAC as per PDAF data which suggested that all 26 participants with cardiologist-confirmed cases of ‘possible AF’ qualified for OAC (each participant had a CHA_2_DS_2_-VASc score of 2 or more).
- The proportions of patients initiated on DOAC and VKA therapies were 56% and 44%, respectively as indicated by the percentages of patients receiving each therapy during the PDAF study.

Both patient cohorts entered the Markov simulation model in the health state of ‘stable AF’ and over time transitioned into either the state of ‘post-stroke’ (post-ischaemic stroke), ‘post-major bleed’ or ‘death’ as shown in the S1C_Fig. Patients were followed up for a period of 10 years and were allowed to transition between the health states every 3 months as in the cost-effectiveness study by Jacobs *et al.* [4] and the systematic review by Welton et al.[9] Transition probabilities between the health states were estimated using the data from landmark clinical trials of OAC [12-19] and were adjusted for mortality rates in individuals aged 65 years and above, across England and Wales [5, 6]. The utilities of health states were derived from the Dutch model by Jacobs *et al*. [4] and varied from 0.84 for stable AF to 0.45 and 0.67 following ischaemic stroke and major bleed, respectively. The rates of clinical events/mortality were assumed to be constant over time. The future health gains and costs were discounted by 1.5% and 3.5%, respectively as recommended by NICE [20].

The probabilistic sensitivity analysis (PSA) employed a Monte Carlo simulation of the Markov model generating 100,000 pseudorandom incidence rates as previously described [3]. The costs (S1B-D_Tables) were varied between 50% and 150% of the base case. The screening participation rate was varied from 30% to 80% (base case 50%) whereas the level of adherence to OAC ranged from 40% to 80% (base case 55%) [3].

**S1C_Fig:** Markov-state diagram used in the cost-effectiveness evaluation of PDAF atrial fibrillation (AF) screening strategy. The diagram displays the health states of Stable AF, Post-stroke, Post-major bleed and Death. It also displays the temporary states of Stroke and Major bleed which lead onto the Post-stroke and Post-major bleed states, respectively. Each transition from one health state to another is accompanied by the transition probability denoted by letter “p”. For the purpose of this evaluation, it was assumed that once patients entered the health states of Post-stroke or Post-major bleed, they might only transition into Death and no other health states. **N.b.** “stroke” includes all incidences of ischaemic stroke.

**S1B_Table**: A summary of input parameters for Markov cohort simulations conducted as part of cost-effectiveness evaluation of PDAF AF screening strategy in general practice surgeries using Kardia Mobile® devices. Transition probabilities and utilities were assumed to follow a beta distribution whereas the costs were assumed to display lognormal distribution. Abbreviations: PDAF – Pharmacists Detecting Atrial Fibrillation; AF – atrial fibrillation; PSA – probabilistic sensitivity analysis; ECG – electrocardiogram.

| **Cost Parameter** | | **Base Case (£)** | **Range in PSA (£)** | | **References** |
| --- | --- | --- | --- | --- | --- |
|  |  |  | Lower | Upper |  |
| 3-monthly AF screening costs/patient | | 286.96 | 143.48 | 430.44 | [2, 7-9, 21, 22] |
| *Cost of single-lead ECG screening** | | *0.90* | *-* | *-* | [7, 21] |
| *Cost of new diagnosis*** | | *127.95* | *-* | *-* | [8, 9, 22] |
| *Cost of oral anticoagulant therapy* | | *158.11* | *-* | *-* | [2] |
| Cost of ischaemic stroke | | 3,395.08 | 1,697.54 | 5,092.62 |  |
| Cost of major bleed | | 325.73 | 162.87 | 488.60 |  |
| **Transition Probabilities** | | **Base Case** | **Range in PSA** | | **References** |
|  |  |  | Lower | Upper |  |
| Ischaemic stroke from stable AF | No screening | 0.0076 | 0.0075 | 0.0077 | [5, 6, 12-19] |
|  | Screening | 0.0068 | 0.0067 | 0.0070 |  |
| Major bleed from stable AF | No screening | 0.0055 | 0.0053 | 0.0056 |  |
|  | Screening | 0.0058 | 0.0057 | 0.0059 |  |
| Death from stable AF | No screening | 0.0362 | 0.0360 | 0.0365 | [5, 6, 12-19] |
|  | Screening | 0.0351 | 0.0349 | 0.0354 |  |
| Death from ischaemic stroke | No screening | 0.1340 | 0.1282 | 0.1399 | [4-6, 12-19] |
|  | Screening | 0.1300 | 0.1240 | 0.1361 |  |
| Death from major bleed | No screening | 0.0543 | 0.0498 | 0.0589 | [5, 6, 12-19, 23] |
|  | Screening | 0.0527 | 0.0484 | 0.0571 |  |
| **Utilities** | | **Base Case** | **Range in PSA** | | **References** |
|  |  |  | Lower | Upper |  |
| Stable AF | | 0.8430 | 0.7587 | 0.9273 | [4] |
| Post-ischaemic stroke | | 0.4490 | 0.3610 | 0.5370 |  |
| Post-major bleed | | 0.6660 | 0.5355 | 0.7965 |  |

**S1C_Table**: A summary of input parameters for Markov cohort simulations conducted as part of the cost-effectiveness evaluation of PDAF AF screening strategy in general practice surgeries using pulse palpation. Transition probabilities and utilities were assumed to follow a beta distribution whereas the costs were assumed to display lognormal distribution. Abbreviations: PDAF – Pharmacists Detecting Atrial Fibrillation; AF – atrial fibrillation; PSA – probabilistic sensitivity analysis; ECG – electrocardiogram.

| **Cost Parameter** | | **Base Case (£)** | **Range in PSA (£)** | | **References** |
| --- | --- | --- | --- | --- | --- |
|  |  |  | Lower | Upper |  |
| 3-monthly AF screening costs/patient | | 275.15 | 131.67 | 418.63 | [2, 7-9, 21, 22] |
| *Cost of screening** | | *0.87* | *-* | *-* | [7, 21] |
| *Cost of new diagnosis*** | | *116.17* | *-* | *-* | [8, 9, 22] |
| *Cost of oral anticoagulant therapy* | | *158.11* | *-* | *-* | [2] |
| Cost of ischaemic stroke | | 3,395.08 | 1,697.54 | 5,092.62 |  |
| Cost of major bleed | | 325.73 | 162.87 | 488.60 |  |
| **Transition Probabilities** | | **Base Case** | **Range in PSA** | | **References** |
|  |  |  | Lower | Upper |  |
| Ischaemic stroke from stable AF | No screening | 0.0076 | 0.0075 | 0.0077 | [5, 6, 12-19] |
|  | Screening | 0.0070 | 0.0068 | 0.0071 |  |
| Major bleed from stable AF | No screening | 0.0055 | 0.0053 | 0.0056 |  |
|  | Screening | 0.0058 | 0.0056 | 0.0059 |  |
| Death from stable AF | No screening | 0.0362 | 0.0360 | 0.0365 | [5, 6, 12-19] |
|  | Screening | 0.0353 | 0.0351 | 0.0356 |  |
| Death from ischaemic stroke | No screening | 0.1340 | 0.1282 | 0.1399 | [4-6, 12-19] |
|  | Screening | 0.1307 | 0.1247 | 0.1367 |  |
| Death from major bleed | No screening | 0.0543 | 0.0498 | 0.0589 | [5, 6, 12-19, 23] |
|  | Screening | 0.0530 | 0.0486 | 0.0574 |  |
| **Utilities** | | **Base Case** | **Range in PSA** | | **References** |
|  |  |  | Lower | Upper |  |
| Stable AF | | 0.8430 | 0.7587 | 0.9273 | [4] |
| Post-ischaemic stroke | | 0.4490 | 0.3610 | 0.5370 |  |
| Post-major bleed | | 0.6660 | 0.5355 | 0.7965 |  |

*The cost of screening per individual in England and Wales [5, 6] included the clinical pharmacist’s time (11 min/appointment) and the acquisition cost of 6,000 Kardia Mobile^®^ devices (for device-based model only) [24].

**The cost of a new AF diagnosis was based on the prevalence of previously undiagnosed AF (1.3%) determined by the Cardiologist’s interpretation of single-lead ECG and the sensitivity of the index test for the identification of AF. It took into account the cost of 12-lead ECG procedures and associated GP interpretations following the initial referral as well as the cost of GP and Cardiologist’s appointments for new AF diagnoses (10 minutes each). It also considered the hypothetical costs of extra 12-lead ECGs and GP interpretations which would have been incurred due to false positive AF and unclassified/unreadable diagnoses resulting from the device’s algorithm or pulse palpation. Based on the follow-up data, this economic model assumed that 76% of those with unclassified/unreadable diagnoses would be followed up with a 12-lead ECG and a GP interpretation.

**S1D_Table**: A breakdown of costs used in the cost-effectiveness evaluation of PDAF AF screening strategy. Abbreviations: ECG – electrocardiogram; AF – atrial fibrillation; GP – general practitioner.

| Unit | Cost/Unit (£) | Reference |
| --- | --- | --- |
| Kardia Mobile^®^ device | 99.00 | [21] |
| AF screen by clinical pharmacist (Agenda for Change Band 7, 11 min) | 3.49 | [7] |
| 12-lead ECG and GP review of ECG | 39.95 | [22] |
| GP appointment for new diagnosis (10 min) | 22.43 | [9] |
| Cardiologist appointment for new diagnosis (10 min) | 23.82 | [9] |
| Warfarin annual acquisition | 45.89 | [2] |
| Rivaroxaban annual acquisition | 851.27 |  |
| Apixaban annual acquisition | 890.36 |  |
| Anticoagulation clinic for warfarin (annual) | 268.25 |  |
| Ischaemic stroke | 13,580.30 |  |
| Major bleed | 1,302.92 |  |

**References**

1. Edlin R, McCabe C, Hulme C, Hall P, Wright J. Cost Effectiveness Modelling for Health Technology Assessment: A Practical Course. London: Adis; 2015.

2. National Institute for Health and Care Excellence. Costing Report: atrial fibrillation implementing the NICE guideline on atrial fibrillation (CG180) [Internet]. 2014 [cited 2020 January 14]. Available from: <https://www.nice.org.uk/guidance/cg180/resources/costing-report-pdf-243730909>.

3. Lowres N, Neubeck L, Salkeld G, Krass I, McLachlan AJ, Redfern J, et al. Feasibility and cost-effectiveness of stroke prevention through community screening for atrial fibrillation using iPhone ECG in pharmacies. The SEARCH-AF study. Thromb Haemost. 2014;111(6):1167-76. doi: 10.1160/th14-03-0231.

4. Jacobs MS, Kaasenbrood F, Postma MJ, van Hulst M, Tieleman RG. Cost-effectiveness of screening for atrial fibrillation in primary care with a handheld, single-lead electrocardiogram device in the Netherlands. Europace. 2018;20(1):12-8. doi: 10.1093/europace/euw285.

5. Office for National Statistics. Deaths registered in England and Wales: 2016 [Internet]. 2016 [cited 2020 January 14]. Available from: <https://www.ons.gov.uk/peoplepopulationandcommunity/birthsdeathsandmarriages/deaths/bulletins/deathsregistrationsummarytables/2016#links-to-related-statistics>.

6. Office for National Statistics. Mortality statistics - underlying cause, sex and age [Internet]. 2017 [cited 2020 January 14]. Available from: <https://www.nomisweb.co.uk/query/construct/summary.asp?mode=construct&dataset=161&version=0>.

7. NHS Employers. NHS Terms and Conditions (AfC) pay scales - Hourly [Internet]. 2019 [cited 2020 January 14]. Available from: <https://www.nhsemployers.org/pay-pensions-and-reward/agenda-for-change/pay-scales/hourly>.

8. NHS Improvement. National tariff payment system 2017/18 and 2018/19 [Internet]. 2017 [cited 2020 January 14]. Available from: <https://improvement.nhs.uk/resources/national-tariff-1719/#h2-tariff-documents>.

9. Welton NJ, McAleenan A, Thom HH, Davies P, Hollingworth W, Higgins JP, et al. Screening strategies for atrial fibrillation: a systematic review and cost-effectiveness analysis. Health Technol Assess. 2017;21(29):1-236. doi: 10.3310/hta21290.

10. NICE. The guidelines manual [Internet]. 2012. Available from: <https://www.nice.org.uk/process/pmg6/resources/the-guidelines-manual-pdf-2007970804933>.

11. Kirchhof P, Benussi S, Kotecha D, Ahlsson A, Atar D, Casadei B, et al. 2016 ESC Guidelines for the management of atrial fibrillation developed in collaboration with EACTS. Eur Heart J. 2016;37(38):2893-962.

12. Granger CB, Alexander JH, McMurray JJV, Lopes RD, Hylek EM, Hanna M, et al. Apixaban versus Warfarin in Patients with Atrial Fibrillation. N Engl J Med. 2011;365(11):981-92. doi: 10.1056/NEJMoa1107039.

13. Connolly SJ, Ezekowitz MD, Yusuf S, Eikelboom J, Oldgren J, Parekh A, et al. Dabigatran versus Warfarin in Patients with Atrial Fibrillation. N Engl J Med. 2009;361(12):1139-51. doi: 10.1056/NEJMoa0905561.

14. Patel MR, Mahaffey KW, Garg J, Pan G, Singer DE, Hacke W, et al. Rivaroxaban versus Warfarin in Nonvalvular Atrial Fibrillation. N Engl J Med. 2011;365(10):883-91. doi: 10.1056/NEJMoa1009638.

15. Connolly SJ, Laupacis A, Gent M, Roberts RS, Cairns JA, Joyner C. Canadian Atrial Fibrillation Anticoagulation (CAFA) Study. J Am Coll Cardiol. 1991;18(2):349-55.

16. Ezekowitz MD, Bridgers SL, James KE, Carliner NH, Colling CL, Gornick CC, et al. Warfarin in the prevention of stroke associated with nonrheumatic atrial fibrillation. Veterans Affairs Stroke Prevention in Nonrheumatic Atrial Fibrillation Investigators. N Engl J Med. 1992;327(20):1406-12. doi: 10.1056/nejm199211123272002.

17. Petersen P, Boysen G, Godtfredsen J, Andersen ED, Andersen B. Placebo-controlled, randomised trial of warfarin and aspirin for prevention of thromboembolic complications in chronic atrial fibrillation. The Copenhagen AFASAK study. Lancet. 1989;1(8631):175-9.

18. EAFT Study Group. Secondary prevention in non-rheumatic atrial fibrillation after transient ischaemic attack or minor stroke. EAFT (European Atrial Fibrillation Trial) Study Group. Lancet. 1993;342(8882):1255-62.

19. Mcbride R. Stroke Prevention in Atrial-Fibrillation Study - Final Results. Circulation. 1991;84(2):527-39.

20. NICE. Methods for the development of NICE public health guidance (third edition) [Internet]. 2012. Available from: <https://www.nice.org.uk/process/pmg4/chapter/incorporating-health-economics>.

21. Alivecor. Kardia Mobile [Internet]. 2019 [cited 2020 January 14]. Available from: <https://shop.gb.alivecor.com/>.

22. National Institute for Health and Care Excellence. AliveCor Heart Monitor and AliveECG app (Kardia Mobile) for detecting atrial fibrillation [Internet]. 2015 [cited 2020 January 14]. Available from: <https://www.nice.org.uk/advice/mib35/chapter/technology-overview>.

23. Eikelboom JW, Mehta SR, Anand SS, Xie C, Fox KA, Yusuf S. Adverse impact of bleeding on prognosis in patients with acute coronary syndromes. Circulation. 2006;114(8):774-82. doi: 10.1161/circulationaha.106.612812.

24. The AHSN network. Atrial Fibrillation: detect, protect and perfect [Internet]. 2017 [cited 2020 January 14]. Available from: <https://www.ahsnnetwork.com/about-academic-health-science-networks/national-programmes-priorities/atrial-fibrillation>.
