# Supplementary material for: Opportunistic screening for atrial fibrillation by clinical pharmacists in UK general practice during the influenza vaccination season: A cross-sectional feasibility study
Source: PLoS Med. 2020 Jul 17;17(7):e1003197. doi: 10.1371/journal.pmed.1003197 (PMC7367445; doi:10.1371/journal.pmed.1003197)
Supplement: S1 Appendix — (PDF) [file pmed.1003197.s003.pdf]

Atrial Fibrillation Screening in General Practice by Clinical Pharmacists

Participant Questionnaire

Please complete this questionnaire to tell us about your appointment with the clinical Pharmacist today for a **pulse check and ECG**.

Your feedback will enable us to identify areas that may need improvement. Your opinions are therefore very valuable.

There are no right or wrong answers. We are interested in your honest views.

By taking part you are giving your consent for your answers to be used as described in the patient information leaflet, that you received before having your appointment. It is important for you to know that your Practice will not know whether you have participated in the questionnaire or not, and that taking part will not affect your future care in any way.

For all queries regarding this study, please do not hesitate to contact:

Research Lead:

Instructions: Please answer all the questions on the following pages. Please tick or mark the box that best reflects your level of agreement. Please **indicate one response only** for each statement.

We think that it will take you about **5 minutes** to complete this questionnaire.

Thank you

**Practice Name:**		
**Date of Visit:**	____________ [dd/mm/yyyy]	

1. Your GP practice was part of a screening study that supports the early detection and diagnosis of atrial fibrillation. From your experience of it, how important was the screening for you?
   ☐ **Very Important** ☐ **Important**
   ☐ **Not Important**

2. Were you aware of this condition before you were screened?
   ☐ **Aware** ☐ **Not Aware**

3. Were you aware of any of the health risks associated with this condition, before you were screened?
   ☐ **Aware** ☐ **Not Aware**

4. How satisfied were you with the information provided before the appointment?
   ☐ **Very Satisfied** ☐ **Satisfied** ☐ **Dissatisfied** ☐ **Very Dissatisfied**

5. Did the Pharmacist clearly explain what was involved by having your pulse tested?
   ☐ **Yes** ☐ **No**

6. Did the Pharmacist clearly explain what is involved in having an ECG?
   ☐ **Yes** ☐ **No**

7. Afterwards did the Pharmacist clearly explain the results of the test to you?
   ☐ **Yes** ☐ **No**

8. How satisfied were you with the information provided after the appointment?
   ☐ **Very Satisfied** ☐ **Satisfied** ☐ **Dissatisfied** ☐ **Very Dissatisfied**

9. Please rate how well you thought the Pharmacist carried out the tests:

☐ **Very Good** ☐ **Good** ☐ **Poor** ☐ **Very Poor**

10. Did the Pharmacist make you feel at ease? ☐ **Yes** ☐ **No**

11. How satisfied were you with the length of the appointment?

☐ **Very Satisfied** ☐ **Satisfied** ☐ **Dissatisfied** ☐ **Very Dissatisfied**

12. Overall how satisfied were you with the service that you received?

☐ **Very Satisfied** ☐ **Satisfied** ☐ **Dissatisfied** ☐ **Very Dissatisfied**

13. If the test was offered to you again next year, would you have it done?

☐ **Yes** ☐ **No**

14. Was there anything you particularly **LIKED** about the service?

☐ **Yes** ☐ **No**

If you particularly liked something about the service please tell us what it was and what was good about this?

15. Was there anything that you particularly **DISLIKED** about the service?

☐ **Yes** ☐ **No**

If you particularly disliked something, please tell us about this.

16. Would you be happy to see the Pharmacist for **other screening tests** in the future?

☐ **Yes** ☐ **No**

What other screening tests would you like to receive from a Pharmacist?

17. Please add any further comments that may help us to **improve** this proposed AF screening strategy.

Further comments:

Thank you for taking the time to complete this questionnaire.
