# Supplementary material for: Opportunistic screening for atrial fibrillation by clinical pharmacists in UK general practice during the influenza vaccination season: A cross-sectional feasibility study
Source: PLoS Med. 2020 Jul 17;17(7):e1003197. doi: 10.1371/journal.pmed.1003197 (PMC7367445; doi:10.1371/journal.pmed.1003197)
Supplement: Attachment — Submitted filename: Response to reviewers.pdf [file pmed.1003197.s008.pdf]

Requests from the editors:

1. The reviewers noted that your manuscript is possibly too long – please try to condense your findings more concisely. Additionally, the reviewers raised concerns over the usefulness of the economic evaluation presented in your study. While you do not necessarily need to remove the economic data altogether, I would strongly advise that you present these results in a manner more concise, and more relevant to your main conclusions.

As requested by editors and reviewers we have tried to make the economic evaluation of the study more concise and relevant to the study. We have moved the detailed methodology and results to a supplementary file (S1 Supporting Information) and condensed the text in the manuscript.

2. Please revise your title according to PLOS Medicine's style. Your title must be nondeclarative and not a question. It should begin with main concept if possible. "Effect of" should be used only if causality can be inferred, i.e., for an RCT. Please place the study design ("A randomized controlled trial," "A retrospective study," "A modelling study," etc.) in the subtitle (ie, after a colon).

We have made two changes to the title. As was requested by reviewer 1 (point 2) we have removed systematic population and replaced it with opportunistic and in accordance with PLOS medicine's style we have included "a cross-sectional feasibility study".

Opportunistic screening for atrial fibrillation by clinical pharmacists in general practice during the influenza vaccination season: a cross-sectional feasibility study.

3. In your Abstract, please include some overall demographical data for your patients, eg. age, sex.

Thank you to the editors for this request. We have added the details of participants' age and sex to the 'Methods and Findings' section of the abstract (page 2, Line 29) as follows:

"604 participants were screened **(median age 73 years, 42.7% male)**."

4. Please ensure that all corresponding p values are provided for all quantitative results in the abstract and throughout your manuscript.

We have double checked that all corresponding p values have been provided for quantitative results.

5. In Table 1, we ask that you expand the Ethnicity: "Other" category to show more granularity and to avoid any possible perceptions of marginalisation.

Thank you for the request to expand this demographic category. We have revised Table 1 to include additional ethnicities and provided the breakdown of the remaining participants in a form of two footnotes as follows:

"*White European, Flemish, Italian, Scottish and South African (n = 1 each), and White Non-specified or Other (n = 2). **Kazakh, American, Australian, Hungarian and Norwegian (n = 1 each)."

6. At this stage, we ask that you include a short, non-technical Author Summary of your research to make findings accessible to a wide audience that includes both scientists and non-scientists. The Author Summary should immediately follow the Abstract in your revised manuscript. This text is subject to editorial change and should be distinct from the scientific abstract. Please see our author guidelines for more information: https://journals.plos.org/plosmedicine/s/revising-your-manuscript#loc-author-summary

We have included an Author Summary

7. Please reiterate a brief description of the content analysis approach cited on line 201.

We have amended this section to include a brief description of the content analysis approach as follows:

"Responses to open-ended questions of participant experience questionnaires were imported into Nvivo (V.12) and analysed using content-analysis [36], a systematic approach commonly applied to the analysis of verbatim questionnaire data [37]. This included coding the words and frequencies extracted from the questionnaires to identify the frequency of their occurrence and to group them into key themes. The themes were considered alongside responses to closed questions."

We have also amended the 'Quantitative Data Analysis' section to indicate the analysis of participant answers to closed questions of the questionnaire as follows:

"Categorical variables, including responses to closed questions of the participant experience questionnaire, were expressed as numbers and percentages (%)."

8. In your STARD checklist, please use section and paragraph numbers, rather than page numbers.

We have amended the STARD checklist as requested.

9. PLOS Medicine requires that the de-identified data underlying the specific results in a

published article be made available, without restrictions on access, in a public repository or as Supporting Information at the time of article publication, provided it is legal and ethical to do so. Please see the policy at http://journals.plos.org/plosmedicine/s/data-availability and FAQs at http://journals.plos.org/plosmedicine/s/data-availability#loc-faqs-for-data-policy

Thank you to the editors for this request. We have produced three additional supplementary files (S2-S4_Appendix) in Microsoft Excel format, which contain all de-identified data underlying the findings of our study. These files have been re-submitted alongside the revised manuscript.

10. Please provide a copy of the questionnaire used in your study as a Supporting Information file.

The participant questionnaire has been included as a supplementary file (S1_Appendix.PDF) and submitted alongside the manuscript.

11. The usefulness of the word cloud is limited, so we would advise not including it in your manuscript.

As requested, we have removed this figure from the manuscript and removed reference to it from the manuscript.

Comments from the reviewers:

Reviewer #1: This is a manuscript on screening for atrial fibrillation performed by clinical pharmacists during influenza vaccination season in a general practice setting. Individuals attending influenza vaccination aged above 65 were offered examination using pulse palpation and one-lead ECG (Kardia Mobile). Pharmacists were trained in palpating pulse and in recording and interpreting one-lead ECG. Results from pharmacist examinations were compared to ECG interpretation by cardiologist. The manuscript also includes a health economy assessment and results from feedback given by the participants. The authors concluded that the screening procedure was feasible, economically viable and positively endorsed by participants.

Comments:

1. In general, I believe that the manuscript is too voluminous, and the present volume will probably discourage some readers from reading the entirety. I suggest putting the health economy data in a separate paper.

We agree that this was too voluminous, and as requested by the editors, we have made this section much more concise in the manuscript (page 17, from line 341) and have moved the details of the methodology and results to a supplementary file (S1 Supporting Information).

2. The title: If you offer a screening measure to an individual who is attending health care for another reason, the screening is opportunistic, not systematic. The title also implies that this is population screening, but since not all aged above 65 will get the screening invitation, I am reluctant to describe this screening effort as population-based.

We have amended the title from "Systematic population screening" to "**Opportunistic screening**" as per your recommendation.

3. Abstract: even though feasibility is the primary outcome, many readers would like to find data on the screening yield as well in the abstract.

Thank you to the reviewer for requesting additional information about the screening yield in the abstract. We have added the following sentence to the abstract (page 2, line 34) accordingly:

"At follow-up, four participants (0.7%) were diagnosed with new AF and 3 (0.5%) were initiated on anticoagulation."

4. I would designate the Kardia ECG device digital technology but hardly novel.

As requested we have removed the word "novel" from the text when referring to the digital technology

5. It is reported that 604 patients were screened, but how many were offered screening in total and how many declined? This is core data when judging a screening program. Furthermore, if there were data on the number of inhabitants aged over 65 in the catchment areas one could estimate the proportion of the population reached by this screening effort. As a suggestion, the boxes in figure 1 should contain numbers reporting the loss of participants at different stages.

Due to the nature of the recruitment process in this particular study and because we did not attend every flu clinic in each of the participating practices, we did not have a systematic way of recording how many patients were offered or could have been offered AF screening at each practice and who then subsequently declined. We agree that this would have been useful information to collect if all of the patients attending these practices were being offered screening.

6. Introduction, line 56: Hypertension and heart failure are more common co-morbidities in AF.

Thank you, we have adjusted the text accordingly, (page 3, line 57).

7. Clinical pharmacists and cardiologists were not blinded from knowing the ECG interpretation made by the Kardia Mobile algorithm. This could have affected their ECG interpretation and is a major limitation of the validation part of the study and should be recognised as such.

During the development of the screening protocol, we made the collective decision with the cardiologist to leave the algorithm switched on. One reason for this was so that we could test the accuracy, specificity and sensitivity of the device, compared with the cardiologist. The cardiologist also felt that the device was designed to be used with its' algorithm and so ultimately in any clinical setting, this would be how the device would be used and should be tested. We agree that having the algorithm switched on may have influenced the diagnostic decision of the pharmacists and as such that this was a limitation of the study design and have thus increased the emphasis of this point in the "strengths and limitation" section of the manuscript (page 22, line 444) as shown below:

"Measuring the accuracy of the pharmacist to detect AF using the $_{SL}$ECG was limited by the protocol design, where the automated algorithm was retained on the KMD. The interpretation of the $_{SL}$ECG by the algorithm may have influenced the diagnostic decision made by the

pharmacist potentially increasing the risk of misclassification bias, and thus the level of diagnostic accuracy observed may not be truly representative of their capability."

8. Why was not medical history collected from all participants?

During the design of the protocol we were extremely conscious of minimising any associated workload burden that this study may incur on extremely busy GP practices. As such we made the decision to only collect enhanced medical history from those participants identified with "AF" or "other cardiovascular complications" and a random sample of the "normal" participants.

9. This study was intended to validate the use of clinical pharmacists in AF screening and their ECG interpretation as compared to ECG interpretation made by a cardiologist. Since the absolute precision is depending on prevalence of atrial fibrillation, which was low in the screened population, one must consider sample size, i.e. what is the absolute precision in the validation trial with the reported sample size and AF prevalence?

We agree that the prevalence of AF is low in the screened population and variable in other published estimates. Together with the occurrence of paroxysmal AF, this makes it difficult to obtain an accurate measure of precision. To mitigate this, for the purposes of evaluation and comparison, we defined the cardiologist's diagnosis as being 100% accurate. As such, we designed the study to ensure we obtained sufficient suspected AF cases for this comparison to be valid. Using the lowest prevalence of 0.05 (5%) a sample size of 600 would be expected to generate 30 cases of suspected AF (lower 95% CI of 20). In this study, the cardiologist diagnosed 26 cases.

10. Table 1: A percent sign after percentages would increase readability. Abbreviations m and bpm should be explained.

Thank you to the reviewer for the request to improve the readability of Table 1. We have added the percent signs after the percentages of each categorical variable. We have also provided the explanations for abbreviations as follows:

"Abbreviations: BMI – body mass index; bpm – beats per minute (heart rate)."

The abbreviations of m^2, cm and kg were not explained in full considering their inclusion as the standard abbreviations of units of measurement in the International System of Units (SI).

11. It is reported that only 1% of single lead ECG recordings were deemed as uninterpretable

by a cardiologist. This is a very low proportion as compared to previous studies. Were there in some cases multiple recordings performed to achieve acceptable signal quality?

Thank you for requesting to clarify the number of ECG recordings performed for each participant. In order to address this comment, we added the following information to the Results section (page 11, line 236):

"Nearly 85% of participants only had one $_{SL}$ECG recording (512/604), although two or more ECGs were performed in 15.2% of participants (92/604) where the first recording was of poor quality."

12. Paragraph "SLECG interpretation by the clinical pharmacists" starting on line 307: Many of the results in the text are duplicated from table 2, this should be avoided. The Cohens kappa coefficient should be added to table 2.

We have added the details of Cohen's Kappa statistic with respective 95% confidence intervals to Table 2 and removed duplicated results of Table 2 from the text, as requested.

13. Paragraph "AF prevalence" starting on line 342: I have difficulties following the figures reported here. It is stated that 18/26 patients had known AF, which implies that the remaining 8 were identified from screening. It is stated on line 345 that 4/604 new cases were diagnosed within the screening project. The figures in the upper part of this paragraph should be revised and clarified. How many participants were diagnosed with new AF?

We have reviewed and clarified the figures to distinguish between the prevalence of new AF determined by the cardiologist's interpretation of $_{SL}$ECG and by the follow-up confirmation through $_{12L}$ECG as follows (page 15 line 306):

"The total prevalence of 'known' and 'new' AF ascertained by the cardiologist's interpretation of $_{SL}$ECG recordings was 4.3% (26/604). Of these 26 participants, 18/26 (2.9%) had a known medical history of AF, were in AF when screened and no further action was taken. A total of 8 (1.3%) possible-AF participants were referred for a $_{12L}$ECG. Three (0.5%) of these referred participants remained in AF at time of the $_{12L}$ECG. In total, 4/604 (0.7%) participants were diagnosed with 'new' AF as a result of screening after a $_{12L}$ECG confirmation (3 with initially suspected 'possible AF' and 1 with an 'unclassified' diagnosis)."

14. In the same paragraph, it is stated on line 349 that all 26 "actionable" AF participants were eligible for OAC treatment and that 20/26 were on this treatment at the end of the study. How many of these 20 treated patients had known AF and newly diagnosed AF? How many of the patients with known AF (n=18) had OAC treatment on study entry? This figure is vital for the health economy assessment.

We have revised the paragraph (Page 15, Lines 312) to indicate the proportions of participants with 'known' and 'new' AF who were anticoagulated as follows:

"Interestingly, of the 18 'known' AF patients, all of whom were receiving OAC treatment, only seven reported at the time of screening, that they suffered from AF and were receiving anticoagulation therapy and 3 participants were unsure about their diagnosis or treatment warranting a confirmation in their medical records. "

and

"Of the 26 participants eligible for OAC therapy, 20 (76.9%) were on OAC therapy, at the end of the study (18 with 'known' and 2 with 'new AF'). An additional participant with a provisional 'unclassified' diagnosis who was diagnosed with 'new' AF following a $_{12L}$ECG was anticoagulated accordingly."

15. Line 368: the statistical methods used should only be reported in the methods section.

We have removed the details of statistical tests used to estimate the between-group differences reported in Table 3 as requested. The details of statistical tests used are now only available in the Methods section (page 8, line 170).

16. Table 5: Many of the ECG conditions described in table 5 (BBB, low-grade AV-block) will not lead to additional investigations in asymptomatic patients. The therapeutic implication is very different in low-grade and in high-grade AV-block, these should be classified. Were any participants referred for pacemaker implantation?

Thank you to the reviewer for their request to clarify the follow-up diagnostic data presented in Table 5 (now Table 4). All eight participants who were diagnosed with an AV block at follow-up were confirmed to have the first-degree AV block which did not require additional investigations or treatment. Although the detailed investigations and treatment of non-AF conditions were beyond the scope of this study, none of the participants were referred for pacemaker implantation. To clarify the degree of the AV block, we have amended the abbreviations list for Table 4 as follows:

"AVB – first-degree atrioventricular block"

We have also added a sentence to the results section (page 18, Line 369) as follows:

"None of the participants with a new first-degree AVB (1.3%, 8/604) were referred for pacemaker implantation."

17. Discussion, line 473: there are no data provided supporting the statement that patients with "possible cardiovascular complications", i.e ECG deviations, have a benefit from the screening procedure.

We did identify a number of patients (Table 4) with previously undiagnosed cardiovascular complications that would not have been detected by pulse alone, some of which just required monitoring, others which required treatment or an adjustment of their current treatment. Although not a key focus of our study, we felt that this was an important benefit of using the device. We have adjusted the statement below (page 21, line 424, which we hope will be considered more appropriate.

"Using a $_{SL}$ECG device such as the KMD, which provides a recorded "snap-shot" of a person's heart rhythm was not only beneficial to patients with possible AF, but may also help patients that have other previously undetected cardiovascular complications requiring new treatment or treatment adjustment, for instance heart failure or sinus bradycardia."

18. Discussion, line 507: Given the median age of 73 years of the studied population, I don´t find an AF prevalence of 3.6% high, it is surprisingly low. In the Swedish STROKESTOP 1 trial (Svennberg et al, Circulation 2015:131;2176-84), the baseline prevalence of AF was 9.3% among participants in a population aged 75 and 75.

The difference in prevalence reflects the difference in the screening strategy between the two studies. A single time-point study such as our own, will only likely detect those patients that are in permanent or persistent AF, whilst intermittent monitoring as was done in the STROKESTOP trial, performed over a prolonged period of time, increases the opportunity to detect those patients with paroxysmal AF (PAF). Indeed, this is reflected in our data, where 7/100 of patients that had screened normal at the time of screening were then identified from their medical records as having suffered/suffering from AF/PAF. We have extended our discussion (page 23, line 465) to include the findings of the STROKESTOP study:

"This suggests that the true prevalence of AF in this population is actually higher than is stated and could potentially be as high as 12.3%, as was found in the STROKESTOP study [55]."

19. Discussion, line 525: I don´t think that there is enough data presented to appoint this protocol to the "optimal screening strategy", I suggest removal of this sentence.

We have removed reference to our study data and adapted the sentence, as we feel that the many of the participants that attend flu vaccinations have risk factors that overlap with those for AF and so we feel that this is a viable place to screen people for AF.

"Targeted AF screening of the older population, suffering from one or more risk factors, that overlap with the medical indications recommended for the influenza vaccination [44], would likely make this a viable screening strategy."

20. Conclusion: this paragraph is far too long. I suggest shortening it to 5-10 lines.

As suggested we have reduced the conclusion to the length suggested.

Reviewer #2: Thank you for the opportunity to read and review your manuscript "Systematic population screening for atrial fibrillation by clinical pharmacists in general practice during the influenza vaccination season: the PDAF study". The manuscript is of interest particularly to readers engaged in AF screening, as well as those interested in the involvement of pharmacists in public health initiatives and medical practice.

GENERAL COMMENT
The text is somehow repetitive and written in a verbose style. Much of this could be improved by rephrasing and possibly move some parts to an additional Supplementary document. I.e. details about the participants experience questionnaire; details about how the screening was perceived, which are mentioned in Results and repeated in the Discussion. Furthermore, much results are presented in detail both in the text and in Tables, which may be unnecessary for all numbers.

The manuscript has been considerably shortened and many of the results presented in the tables have been removed from the text. The economic evaluation has been considerably shortened and detailed methodology moved to a supporting information file (S1 Supporting Information).

MAJOR COMMENTS

The manuscript seems long, as it is perceived by this reviewer. A full word count is not available as far as I can see, and can not be retrieved from the available PDF. I trust the Editors may comment on the length of the manuscript with regard to the requirements of the journal, but also for the readability, I would strongly suggest shortening. For example, the Introduction may benefit from shortening, such as the section from page 3, line 64 to page 5, line 92, largely desribes (and discusses) challenges within the UK primary care system. This could be shortened to a few sentences with regard to the focus of this manuscript.

We have considerably shortened the manuscript, in particular we have substantially shortened the section of the introduction as recommended above (page 4 line 65).

I think parts of the Methods description, which are very well described in detail, is presented almost like a protocol, and parts of this could, for the benefit of the reader, be moved to a Supplementary document. For example; page 8, line 160-167 (participants experience questionnaire) could be shortened. Instead of details about the questionnaire, 1-2 sentences could remain in the manuscript, and even more could be included in a Supplementary (even consider writing out in full the questions asked).

We have made the questionnaire available as a supplementary file (S1_Appendix) and have revised the Methods section in relation to the feedback questionnaire as follows (page 8, line 164):

"At the end of the screening appointment all participants were asked to complete a short, anonymous, patient experience questionnaire consisting of 13 closed and 4 open questions (S1_Appendix.pdf) and were offered the opportunity to take part in future focus groups (reported elsewhere). Completed questionnaires were handed over to the receptionist or posted back to the research team using pre-paid envelopes."

The first parts of the Results section are clearly presented. However, you may consider shorten down some of the results repeatedly presented both in the text and in Tables; for example all values for sensitivity, specificity etc. also presented in Table 2.

As suggested, we have removed results from the text that are already presented in Tables.

Furthermore, I would question the relevance of the reported heart rate results (page 15, line 323-327). Although a small but significant difference was found, is this clinical relevant? The manual pulse palpation was followed by the KMD, i.e. not performed simultanously, and this could easily explain the observed difference.

We completely agree and have removed any reference to statistical significance as shown below (page 14, line 294) and removed the reference to the Wilcoxon test from the Methods section.

Average heart rate was determined by the pharmacist to be 70 beats per minute (bpm) [62; 78], compared to 72 bpm [65; 81], n=604, obtained using the KMD.

I would question the validity/relevance of the reported sl-ECG interpretation by the clinical pharmacists, who have (if I understand correctly) just recorded the KMD algorithm interpretation (SR, AF etc.) before recording their own interpretation, which must potentially be biased by the algorithm, reflected in very comparable numbers. Were the 39 cases of "possible AF" (as interpreted by the KMD) the exact same participants as the 39 cases recorded as "possible AF" by the pharmacists? Or did the pharmacists question the automatic algorithm? I can see that this is again further discussed in the Discussion section (page 24, line 494-501). I would suggest to omit these results, or report this as registered "per protocol", but reduce the importance of this. You may also consider rephrasing this in the Discussion. I would assume the pharmacists must have been biased by the KMD (and not as stated now, page 24, line 496-497; that the KMD "...may have influenced the diagnostic decision made by the pharmacist...".

We would like to thank the reviewer for identifying this limitation of study design which has been acknowledged under the 'Strengths and limitations of the study'. In order to emphasise the risk of misclassification bias by the pharmacists following the KMD algorithm we have amended this section (page 22, line 445) as follows:

"The interpretation of the $_{SL}$ECG by the algorithm may have influenced the diagnostic decision made by the pharmacist potentially increasing the risk of misclassification bias, and thus the level of diagnostic accuracy observed may not be truly representative of their capability."

We have also clarified the number of 'possible AF' cases identified by pharmacist's interpretation of $_{SL}$ECG which matched those identified as 'possible AF' by the device's algorithm in the 'Results' section (page 13, line 281) as follows:

“The clinical pharmacists reported 487 (80.6%) cases as normal SR, 39 (6.5%) cases of possible AF (35 of these matched with the KMD algorithm), 71 (11.8%) unclassified and 7 (1.2%) of the ECGs as unreadable.”

This reviewer is not capable of fully assessing the cost-effectiveness evaluation of the screening. However, I would humbly state that I find cost-effectiveness assessments without endpoints (stroke and mortality) troublesome. This is a general comment relevant also for other previous publications regarding AF screening. Although extensive cost-effectiveness analyses have been performed, the costs included/saved in the analysis rely on the basic assumption that screen-detected AF has the same clinical course and risk of stroke, as "ordinary" clinically detected AF. Although this is a likely assumption to make, it is yet to be proven. Furthermore, some numbers in the assumptions made for the "base case", such as a KMD-identified new possible AF rate of 1,3%, is not clear to me. The prevalence of screen-detected AF is 0,7%, and I do not find this number in the analysis. However, this may be right. I am also not fully able to assess how the analysis has taken all other findings (non-AF findings) into account; these may, in a real-life setting, produce a substantial cost for the healthcare system, not to mention unnecessary anxiety and repeated examinations. But, my apologies as I do not have the necessary knowledge to fully assess these analyses.

We would like to thank the reviewer for their comprehensive evaluation of our cost-effectiveness analysis. As mentioned by the reviewer, the Markov cohort simulation model of cost-effectiveness has been commonly employed by other studies evaluating the economic impact of AF screening [2, 3] including the recent systematic review of AF screening strategies commissioned by the National Institute for Health Research [4] and the guidance on the use of single-lead ECG devices to detect symptomatic AF published by the National Institute for Health and Care Excellence (NICE) [5]. Whilst it may not present the endpoints, such as stroke or mortality directly, the Markov simulation model considers such endpoints as the model input parameters when estimating the risk probabilities between the screened and unscreened cohorts [4]. Since each of these endpoints carry an associated cost, this comparison allows for the determination of the incremental cost-effectiveness ratio (ICER) between the cohort of patients with AF which is screened and the cohort of patients with AF which does not engage in screening. This is presented as an incremental cost per quality adjusted life year gained (QALY) and is used by commissioners or policy makers including NICE to assess whether or not the intervention is cost-effective [6].

Indeed, as suggested by the reviewer, all cost-effectiveness evaluations for AF screening to date have been built on the assumption that the screening-detected AF carries the same risk of stroke/mortality as AF detected during routine care. In order to clarify this assumption, we have added the following statement to the list of assumptions about our economic model presented in the supplementary file (S1_Supporting information File):

“Participants with AF identified during the screening were assumed to display the same risk profile of ischaemic stroke and mortality as those with AF incidentally detected during routine care.”

Thank you to the reviewer for their request to clarify the prevalence of ‘new’ AF employed in the cost-effectiveness evaluation. Since the average follow-up time to 12LECG during this study was 16 days, it was likely that some of the participants who were suspected to have a ‘new’ AF following the screening and SLECG interpretation by the cardiologist (1.3%) may no longer be in AF at the time of $_{12L}$ECG (for instance, due to paroxysmal AF). This was witnessed during the study whereby only 3 of the 8 participants (0.5%) with suspected AF remained in AF at the time of their 12LECG (an additional participant with a suspected unclassified diagnosis was confirmed to have AF following a 12LECG making the total prevalence rise to 0.7%). As such, the prevalence of 1.3% determined at the time of screening was employed for the cost-effectiveness model by following the European Society of Cardiology guidance which states that a 30-second ECG strip indicating AF is diagnostic [7]. In order to clarify the prevalence of ‘new’ AF employed by the model, we have amended the supporting information file as follows:

“The costs of screening and new diagnosis of AF per individual were estimated from the total population of individuals aged 65 and above in England and Wales (a population of 10,517,461) [5, 6] assuming the prevalence of new possible AF identified by the cardiologist’s interpretation of single-lead electrocardiogram ($_{SL}$ECG) of 1.3% and the screening participation rate of 50% at base case [3].”

And:

“The prevalence of ‘new’ AF was assumed to be 1.3% based on the cardiologist’s interpretation of $_{SL}$ECG recorded using KMD during the PDAF study. Note that the median time between the screening using KMD and the follow-up confirmatory $_{12L}$ECG was 16.0 [11.0; 24.0] days, and at the time of $_{12L}$ECG, the prevalence of ‘new’ AF was 0.7%. The former value of 1.3% was used for cost-effectiveness analysis to ensure that all participants who may have presented with paroxysmal AF at the time of screening were considered in accordance with the European Society of Cardiology guidance, which indicates that a 30-second ECG recording of AF is diagnostic [11].

MINOR COMMENTS

Page 3, line 46: Reference #2 does not seem to be the most representative publication to document a growing prevalence of AF. Examples of relevant references could be Ball et al (Int J Cardiol, 2013) or Willams et al (Am J Cardiol, 2017).

Page 3, line 48-50: Reference #7 is an industry-initiated "white paper". Although it seems balanced and well written, I would suggest you rather use a comparable relevant and independent publication, such as the white paper "Screening for Atrial Fibrillation" by

Freedman et al (Circulation, 2016), also providing a strong case to improve early detection and screening for AF.

Page 28, line 577: Language-wise: "year by year" instead of "year on year"?

We would like to thank the reviewer for their reference and text suggestions. These changes have been incorporated into the manuscript as requested.

Reviewer #3:
This manuscript evaluated an intervention to perform screenings for atrial fibrillation (AF) during visits to a pharmacist for a routine influenza vaccination. The paper assessed both the accuracy of pharmacists assessments using SL-ECG kardia mobile device and the cost effectiveness of a related screening strategy using a simulation informed by the study. Overall, finding new ways to provide screening for common, but serious, health conditions like AF during provision of routine services is a worthwhile goal. However, I have several concerns about the manuscript.

General comments

1. The two main parts of the study (i.e., the assessment of the accuracy of the screening test and the cost effectiveness study) seem very disconnected. As written, I cannot be certain whether this is simply a disconnect in the way the studies are described or in content.

We have tried to address these points by improving the focus of the manuscript and by moving the detail of the cost effectiveness to a supporting information file (S1 supporting information).

2. The cost effectiveness analysis was difficult to follow and lacked key details (some specific instances are pointed out in the specific comments below). If this component of the study is to be included, it needs to be fleshed out so that the purpose (e.g., what is being compared) as well as the base and alternative scenarios are clear.

Following the request of the editors we have considerably reduced the cost-effectiveness component of the main manuscript and transferred the detailed methodology to a supporting information file (S1 Supporting Information). The comparison between the intervention and alternative scenarios, have been amended in the methods section of the supporting information file (S1 Supporting Information file).

3. The methods section seemed to omit key details related to the assessment of accuracy of the proposed screening approach. Specific instances are referenced in the specific comments below.

We have amended the specific instances referred to below.

4. Restructuring the introduction to highlight the purpose of both components of the study (i.e., the accuracy assessment and the cost effectiveness study) would strengthen the manuscript.

As the cost-effectiveness component of the study has been moved to a supporting information file (S1 Supporting Information), we have not highlighted this in the introduction.

Specific comments

1. Page 5, line 96: should this line read, "we evaluated the use of a single-lead electrocardiogram device *compared with* pulse palpation alone..."?

Manuscript revised accordingly, page 4, line 85.

2. Page 5, line 103: is there a similar checklist or list of standard for reporting results from cost effectiveness studies?

As the main focus of the study was to evaluate the accuracy of pulse palpation and a single-lead ECG device to diagnose AF, with cost-effectiveness being only an outcome from the study, we felt that the STARD checklist was the correct one to use.

3. Page 6, line 121: were pharmacists aware who had pre-existing AF?

Pharmacists were not aware of patients having pre-existing AF prior to screening. Some patients informed them after screening, whilst other patients were aware, they had a problem, but did not know that this condition was AF. We have added a paragraph to explain this in more detail (page 6, line 112):

"Patients with pre-existing AF were not excluded as it was assumed that most participants with AF would self-exclude, whilst those that didn't, would act as positive controls. Pharmacists were unaware of pre-existing AF diagnoses in participants, prior to screening."

4. Page 6, line 124: "consecutive sampling" should be described in more detail. Were all patients who were eligible offered screening? We have expanded the detail of consecutive sampling. (Page 6, line 117):

"Participants were recruited using a consecutive sampling approach, meaning that any participants attending influenza vaccination clinics at participating practices, during the time periods, October 2017 to February 2018 and October 2018 – February 2019, who fulfilled the study inclusion criteria (see above) were invited to participate."

5. Page 6, last paragraph: it seems like there is a sentence or 2 missing here to describe the pharmacists reading of the SL-ECG?

Yes, it does and we have rectified this accordingly (page 6, Line 129):

"The radial pulse of the participant was measured for 60 seconds (s), and this was then followed by a $_{SL}$ECG, recorded for 30 s. Only one ECG was recorded unless the ECG was of poor quality, then a second was recorded. The data of the last ECG recorded for each participant were used for subsequent analysis The ECG was assessed and interpreted by the clinical pharmacist. Their assessment of the ECG was explained to the participant and noted, along with the quality of the ECG recording."

6. Page 8, line 172: what is a "non-parametric" variable?

This is when the population data does not have a normal distribution, we have tried to make this clearer (page 8, Line 173):

"Continuous non-parametric variables, such as those pertaining to demographics, were reported as a median [interquartile range]."

7. Page 8, line 177: When missing data were omitted from the analysis, how was this done? Were participants with any missing data excluded from the entire study or simply excluded from specific analyses where that data point was required?

Data was only excluded from specific analyses to which the missing data point referred; all other data acquired was utilised. We have amended this to make it clearer (page 8, line 178).

"Any missing data points were omitted from final analysis, without data imputation."

8. Page 8, last paragraph: I recommend defining the diagnostic accuracy parameters here (e.g., sensitivity, specificity, positive predictive value, and false discovery rate) here. In addition, the term "accuracy" comes up in the results and is not defined here.

Thank you to the reviewer for their recommendation. We have clarified the definitions of all diagnostic accuracy parameters used as follows (page 9, line 186):

"The sensitivity and specificity of the test were defined as its ability to correctly identify those participants with AF (true positives/true positives and false negatives) and without AF (true negatives/true negatives and false positives), respectively [34]. The overall diagnostic accuracy combined these two parameters as an assessment of the test's ability to detect both the proportions of true positives and true negatives. The PPV and FDR were defined as probabilities that the test will identify those with positive diagnoses either correctly (PPV = true positives/all positives) or incorrectly (FDR = false positives/all positives), respectively [34, 35]."

9. Page 9, line 193: This paragraph was difficult to follow. The first sentence appears to define "diagnoses of AF", but the definition appears to be for AF prevalence. In addition, this paragraph is the first reference (that I see, apologies if I have missed it) to the 12-lead ECG. What is this and what is its purpose in the study?

This sentence has been amended accordingly, (page 9, line 200):

"Prevalence of new AF diagnoses were determined from the number of confirmed AF-positive $_{12L}$ECGs divided by the total number screened with accompanying [95% CI]."

An explanation of the 12-lead ECG was added to the screening procedure (page 7, line 145):

"All patient interventions, including confirmatory 12-lead ECGs ($_{12L}$ECG), were organised by the GP practice in accordance with their normal practice procedures."

10. Page 9, line 201: Here, the manuscript should note the purpose behind analyzing the questionnaire using a content-analysis approach..

Thank you to the reviewer for their request to clarify the selection of the content-analysis approach. We have amended the methods section (page 10, line 206) to provide more information about the content analysis approach used as follows:

"Responses to open-ended questions of participant experience questionnaires were imported into NVivo (V.12) and analysed using content analysis [36], a systematic approach commonly applied to the analysis of verbatim questionnaire data [37]. This included coding the words and frequencies extracted from the questionnaires to identify the frequency of their occurrence and to group them into key themes. The themes were considered alongside responses to closed questions."

We have also amended the 'Quantitative Data Analysis' section (page 8, line 174) to indicate the analysis of participant answers to closed questions of the questionnaire as follows:

"Categorical variables, including responses to closed questions of the participant experience questionnaire, were expressed as numbers and percentages (%)."

11. Page 10, line 210: where does the 1.3% come from in this line? I thought the prevalence of newly identified AF in the study was 0.7%?

Thank you to the reviewer for their request to clarify the prevalence of 'new' AF employed in the cost-effectiveness evaluation. Since the average follow-up time to ${}_{12L}$ECG during this study was 16 days, it was likely that some of the participants who were suspected to have a 'new' AF following the screening and ${}_{SL}$ECG interpretation by the cardiologist (1.3%) may no longer be in AF at the time of ${}_{12L}$ECG (for instance, due to paroxysmal AF). This was witnessed during the study whereby only 3 of the 8 participants (0.5%) with suspected AF remained in AF at the time of their ${}_{12L}$ECG (an additional participant with a suspected unclassified diagnosis was confirmed to have AF following a ${}_{12L}$ECG making the total prevalence rise to 0.7%). As such, the prevalence of 1.3% determined at the time of screening was employed for the cost-effectiveness model by following the European Society of Cardiology guidance which states that a 30-second ECG strip indicating AF is diagnostic [7]. In order to clarify the prevalence of 'new' AF employed by the model, we have amended the methods section of the supporting information file (S1_Supporting Information) as follows:

"The costs of screening and new diagnosis of AF per individual were estimated from the total population of individuals aged 65 and above in England and Wales (a population of 10,517,461) [5, 6] assuming the prevalence of new possible AF identified by the cardiologist's interpretation of single-lead electrocardiogram (${}_{SL}$ECG) of 1.3% and the screening participation rate of 50% at base case [3]."

We have also added an additional clarification of the assumption above in the supporting information file (S1_Supporting information) as follows:

"The prevalence of 'new' AF was assumed to be 1.3% based on the cardiologist's interpretation of ${}_{SL}$ECG recorded using KMD during the PDAF study. Note that the median time between the screening using KMD and the follow-up confirmatory ${}_{12L}$ECG was 16.0 [11.0; 24.0] days, and at the time of ${}_{12L}$ECG, the prevalence of 'new' AF was 0.7%. The former value of 1.3% was used for cost-effectiveness analysis to ensure that all participants who may have presented with paroxysmal AF at the time of screening were considered in accordance with the European Society of Cardiology guidance, which indicates that a 30-second ECG recording of AF is diagnostic [11].

12. Page 10, line 217: The description of the hypothetical screening program needs more detail. Moreover, this sentence makes it unclear if the hypothetical population studied included all those who underwent screening or only those who screened positive and were offered oral anticoagulation medications.

Thank you to the reviewer for this comment. All participants with AF in the intervention cohort were included in the model regardless of whether or not they were identified through screening. In order to clarify this, we have amended the S1_Supporting Information File (from page 6 line 88)

13. Page 10, line 220: What was the purpose of the 3-month cycles? Were these timepoints when outcomes were assessed? This should be clarified.

Three-month cycles are commonly used in the Markov cost-effectiveness simulation [2, 4, 5] and indicate the frequency at which patients within the model are able to transition between the health states, for instance from 'stable AF' to 'post-stroke'. In order to clarify this, we have added further information to the S1_Supporting Information File (page 8, line 136) as follows:

Both patient cohorts entered the Markov simulation model in the health state of 'stable AF' and over time transitioned into either the state of 'post-stroke' (post-ischaemic stroke), 'post-major bleed' or 'death' as shown in the S1C Fig. Patients were followed up for a period of 10 years and were allowed to transition between the health states every 3 months as in the cost-effectiveness study by Jacobs *et al.* [4] and the systematic review by Welton et al.[9]

14. Page 10, line 229: This section of the paper should clearly describe the base cases and alternative scenarios. For example, costs were varied between 50% and 150% of base case, but unclear what these costs are for.

Thank you to the reviewer for their request to clarify the assumptions/costs of the base case and the alternative scenario. We have provided the full breakdown of model assumptions and costs in the S1_Supporting Information File (page 6, Line 88).

15. Page 11, first paragraph: incremental cost effectiveness ratios should be defined here at first use.

We have defined the incremental cost-effectiveness ratios in the S1_Supporting Information File (page 1, Line 19):

The incremental cost-effectiveness ratios (ICERs) were defined as the mean incremental costs divided by the mean incremental quality-adjusted life years (QALYs) when comparing the intervention group and the alternative scenario [9] and were presented as a mean (95% confidence intervals).

16. Table 1: the variables described in this table should be defined in the Methods section, particularly defining how "alcohol drinker" and "positive smoking status" were defined. For example, does this include ever using alcohol or current use only?

We had mentioned this briefly in the methods section, page 6, line 127 and in our previous protocol paper, but have added more detail.

"Participants were asked to complete a basic demographics form (e.g. age, gender, ethnicity, height, weight, current smoking and drinking habits) prior to screening."

We have also amended Table 1 and Table 3 to "Current alcohol drinker" and "Current smoker".

17. Page 15, line 326: is the difference in measured heart rate simply "statistically significant" or is this difference also clinically meaningful? Offering some guidance to readers, here or in the discussion, would be helpful. Line 336

We completely agree that this is not clinically relevant and have removed any reference to statistical significance as shown below (page 14, line 294) and reference to the Wilcoxon test from the Methods section.

Average heart rate was determined by the pharmacist to be 70 beats per minute (bpm) [62; 78], compared to 72 bpm [65; 81], n=604, obtained using the KMD.

18. Page 16, line 351: from this line and the section on follow-up data and outcomes, it appears that there may be a section missing from the methods where follow-up actions should be described.

We have added additional information to the methods, Page 7, line 146:

"All participants given either an AF or unclassified/unreadable diagnosis and a recommendation for further intervention by the cardiologist were followed-up by the study team to ensure participants, had been offered appropriate treatment from their GP practice."

19. Page 18, line 382: what was the threshold used to defined "cost effective" in this study?

We have added the following definition of "cost-effective" with reference to the National Institute for Health and Care Excellence guidance concerning the cost-effectiveness of interventions (page 17, line 347) as follows:

"The intervention was considered to be cost-effective if the estimated ICER was under the willingness-to-pay threshold of £20,000/quality-adjusted life year (QALY) proposed by NICE [43]."

20. Page 18, line 384: It is unclear to me how "participants" were defined here. Was this a cost for all screened individuals, everyone in the population, or some other group?

Thank you to the reviewer for the request to clarify the incremental net benefits presented in the Results. We have added the following definition of the incremental net benefit (INB) to the Methods section of the supporting information file (S1_Supporting Information):

"The incremental net benefit (INB) per patient with new AF identified through screening was calculated as the incremental QALYs multiplied by £20,000 minus the incremental costs [9]."

The INB therefore relates to a patient with new AF in the intervention cohort identified using either KMD or pulse palpation. We have amended the Results section to reflect this definition as follows:

"The INB compared to no screening strategy was £1,903/patient with new AF and £120,084,946/all patients with new AF across England and Wales identified through screening by using KMD, and £946/patient with new AF and £49,741,500/all patients with new AF identified by using pulse palpation."

21. Discussion: some discussion of variability in results between pharmacists would be helpful. Moreover, the discussion would be strengthened by addressing implementation science questions, such as the time required to train pharmacists on the methods needed and whether one would expect accuracy to decline as the intervention was scaled up.

Measuring the variability between individual pharmacists was felt to be beyond the scope of this study, as would have involved all of the pharmacists screening the same patients and was not included as a measured outcome.

22. Page 27, line 551: should this be "... better than for pulse palpation."?

Yes, it should be and have changed accordingly; page 25, Line 501.

References

1. Public Health England. The national flu immunisation programme 2019/20 [Internet]. 2019 [cited 2020 January 14]. Available from: https://www.england.nhs.uk/wp-content/uploads/2019/03/annual-national-flu-programme-2019-to-2020-1.pd.
2. Jacobs MS, Kaasenbrood F, Postma MJ, van Hulst M, Tieleman RG. Cost-effectiveness of screening for atrial fibrillation in primary care with a handheld, single-lead electrocardiogram device in the Netherlands. Europace. 2018;20(1):12-8. doi: 10.1093/europace/euw285.
3. Oguz M, Lanitis T, Li X, Wygant G, Singer DE, Friend K, et al. Cost-Effectiveness of Extended and One-Time Screening Versus No Screening for Non-Valvular Atrial Fibrillation in the USA. Appl Health Econ Health Policy. 2019. doi: 10.1007/s40258-019-00542-y.

4. Welton NJ, McAleenan A, Thom HH, Davies P, Hollingworth W, Higgins JP, et al. Screening strategies for atrial fibrillation: a systematic review and cost-effectiveness analysis. Health Technol Assess. 2017;21(29):1-236. doi: 10.3310/hta21290.
5. NICE. Lead-I ECG devices for detecting symptomatic atrial fibrillation using single time point testing in primary care [Internet]. 2019. Available from: https://www.nice.org.uk/guidance/dg35/resources/leadi-ecg-devices-for-detecting-symptomatic-atrial-fibrillation-using-single-time-point-testing-in-primary-care-pdf-1053752401861.
6. NICE. The guidelines manual [Internet]. 2012. Available from: https://www.nice.org.uk/process/pmg6/resources/the-guidelines-manual-pdf-2007970804933.
7. Kirchhof P, Benussi S, Kotecha D, Ahlsson A, Atar D, Casadei B, et al. 2016 ESC Guidelines for the management of atrial fibrillation developed in collaboration with EACTS. Eur Heart J. 2016;37(38):2893-962.
