# Supplementary material for: Opportunistic screening for atrial fibrillation by clinical pharmacists in UK general practice during the influenza vaccination season: A cross-sectional feasibility study
Source: PLoS Med. 2020 Jul 17;17(7):e1003197. doi: 10.1371/journal.pmed.1003197 (PMC7367445; doi:10.1371/journal.pmed.1003197)
Supplement: Attachment — Submitted filename: Response to requests from Editors and Reviewers - June 2020.pdf [file pmed.1003197.s009.pdf]

Requests from Editors:

1. Please implement the reviewers' final comments. However, please note that you can leave in references in the Discussion section, as long as they are relevant in framing your findings in the context of existing literature. Please see below.

2. Please amend the Title slightly as follows to mention the locale of the study: "Opportunistic screening for atrial fibrillation by clinical pharmacists in UK general practice during the influenza vaccination season: a cross-sectional feasibility study" (no full stop at the end). Amended.

3. Lines 16-17: Please update to: "Growing prevalence of atrial fibrillation (AF) in the ageing population, and its associated life-changing health and resource implications, have led to a need to improve its early detection." – Amended.

4. Line 31: Please define "CHA2DS2-VASc".

We have added:

"...have a CHA_2DS_2-VASc (Congestive heart failure, Hypertension, Age ≥ 75, Diabetes, previous Stroke, Age 65-74 years, Sex category) score ≥ 3."

5. In your abstract and throughout the paper, please quote p values alongside 95% CI, where available.

P values are now quoted where appropriate. Some of the confidence intervals quoted are to illustrate the variability of data and are not used for statistical comparison.

Lines 31-32: We suggest removing the p values. Removed.

We generally ask that exact p values are quoted, except where $p<0.001$, but do not favour quoting them in isolation.

We have amended the necessary p values in the manuscript and added p values as a separate column to Table 3.

6. Line 39, please replace "overwhelmingly" with "generally", or similar.

Amended with: "generally".

7. Please adapt the sentence summarizing study limitations (lines 39-40) to quote one additional limitation, e.g., the limited ethnic representation in the study cohort. We have added (line 41-43):

Key limitations of the study were that the intervention did not reach individuals who did not attend the practice for an influenza vaccination and there was a limited representation of UK ethnic minority groups in the study cohort

8. Please remove the apostrophe from "its" at line 49. Removed

9. Please adapt reference call-outs to the following style: "... health implications [4,5], combined ..." (no space after commas in square brackets).

Reference style has been adapted throughout the manuscript.

10. Line 100: Please give exact date ranges.

The sentence has been amended as follows:

Screening was conducted over two influenza vaccination seasons, from the 28th October 2017 to the 22nd February 2018 and then from the 2nd October to the 14th December 2018.

11. Please remove trademark symbols, e.g. at line 109. Removed from text,

12. Please substitute "sex" for "gender" where appropriate, e.g. at line 128. Replaced.

13. Lines 234 and 236: Please clarify in the text whether the figures in square brackets show range or another statistic.

We have added the range to the text (Line 280):

Median age [interquartile range] of the participants was 73 [69, 78] years and 42.7% of participants were male.

This is also stated in the Methods section and in the legend of Table 1.

14. Table 3: Please give exact p values for all comparisons, including those that are not significant.

We have provided the exact p values in Table 3 as a separate column and updated the expression of p values throughout the manuscript.

15. Table 4: Please replace this with a flowchart, as also recommended by a reviewer. Please also list the abbreviations alphabetically, and include a definition for PR.

We have replaced Table 4 with a simplified flowchart (Fig 3) as requested and listed the abbreviations alphabetically.

16. Lines 387-393: Please provide quantitative data (eg. proportions of patients) to support these statements. We have added in patient numbers to support the statements (Line 443):

In response to open-ended questions, when asked "W*as there anything you particularly liked about the service*?" there were 272 recorded comments. Of these, 75 participants praised the "*professional*" (14), yet "*relaxed*", "*friendly*" and "*at ease*" nature of the pharmacist-led screening (61), while 24 stated that the service improved their access to healthcare by offering an opportunity to obtain a

more rapid provisional diagnosis and reassurance about their health status. 94 participants were particularly pleased with the "*informative*" consultation during which they learnt about AF and where any information was clearly presented in lay terms, they could understand and feel comfortable about and 7 stated that they were particularly impressed with the digital technology that was used. A number of participants (32), particularly liked being able to contribute to clinical research that had a "preventative medicine" focus. When participants were asked "*Was there anything you particularly disliked about the service?"* there were only 6 comments, with length of time of the appointment noted for 2 responses. Finally, when asked "*Would you be happy to see the Pharmacist for other screening tests in the future?*" 112 participants indicated that they would be happy to do this, with the majority (48), willing to engage with any relevant screening tests available.

17. Line 397: Please remove the subjective descriptor "comprehensive". Removed.

18. Line 540: Please avoid stating "effective", as also recommended by a reviewer. Replaced with "feasible"

19. Please correct the link for references 1 and 44. We have amended the links.

20. Author Summary: Addressed all points stated below.

a. Please include the Author Summary in your main manuscript file – after the Abstract, before the Introduction.

b. Bullet point 2: Replace "morbidities" with "conditions" (for clarity to a non-scientific reader).

c. Bullet point 3: Define GP.

Production Issues:

Can you please address the following issues as soon as possible? Your paper has been sent back to enable you to make these changes.

1. Line 65: Please replace "effectively" with "feasibly", or similar. Deleted the prefix.

2. Lines 72, 382, 385, 390: Please hyphenate "cost-effective"/"cost-effectiveness". Hyphenated.

3. Line 415: Please remove the extra semicolon after "electrocardiogram". Removed.

4. Line 453: Please replace "significant" with "notable", or similar. Replaced with notable.

5. Line 547: Please replace" insignificant" with "negligible", or similar. Replaced with negligible.

6. Please correct the link for reference 1 (this still currently points to PLOS submission guidelines). Amended.

7. Please ensure that the funders and grant numbers match between the Financial Disclosure field and the Funding Information tab in your submission form. Note that the funders must be provided in the same order in both places as well. Checked.

8. Please ensure that all of your co-author names match across the article file and the submission form. Please ensure that all author names match verbatim, including the use of a middle name/initials and any special characters (accents, umlauts, etc.), as correct spelling and formatting of author names is vital for accurate indexing in PubMed. Checked.

9. Please ensure that all Supporting Information files use the following format verbatim throughout the manuscript (including legends and all in-text citations): S1 Fig, S1 Table, S1 Text, S1 Data, etc. Each file type should start at 1 and increase numerically. Please note, however, that the file names themselves must use an underscore rather than a space, i.e. S1_Fig.tif, S1_Table.xlsx, S1_Text.doc, S1_Data.xlsx, S1_PRISMA_Checklist.docx, etc.

We have removed the underscore from the in-text citations and added it to the file names themselves.

10. Please ensure that each supporting information file has a legend at the end of the manuscript file, after the Reference list.

We apologise. We had located these files before the references as this is how they had appeared in online manuscripts. We have relocated this list to appear after the references. Each file has a caption. We have added brief legends to some, where appropriate.

11. Please note that your submission cannot contain any references to generic "supporting information" files and instead must refer to specific files using our naming convention (S1 Fig, S1 Table, S1 Text, S1 Data, S1 PRISMA Checklist, etc.)

We have used the description “S1 Supporting Information” as a file name for one of the supporting information files, as it seemed the most appropriate of the examples given (https://journals.plos.org/plosmedicine/s/supporting-information). Please can you confirm that this is a valid item description name, as it does not use “supporting information” in a generic sense?

TABLES:

1. Please ensure that all main tables in their entirety (including titles and footnotes), are placed after the paragraph containing their first in-text citation. Checked and correct

2. Tables 2 and 4 use returns to control text wrapping. This is indicated by a [¶ or ↵] symbol when "Show Paragraph Marks" is turned on in Word. Please either split those cells into

separate cells to achieve alignment or remove the returns. Amended Table 2. Table 4 has been replaced with a new figure (Fig 3).

ARTICLE FILE:

1. Please indicate the corresponding author in the byline by placing an asterisk (*) after their affiliation number. Amended.

2. Please include the corresponding author's email address on the title page of your manuscript, indicated by an asterisk (*). Only an asterisk and the email address itself are required. Amended.

3. Please ensure that all of your co-author names match across the article file and the submission form. Please ensure that all author names match verbatim, including the use of a middle name/initials and any special characters (accents, umlauts, etc.), as correct spelling and formatting of author names is vital for accurate indexing in PubMed. Confirmed.

REFERENCES:

1. Please ensure your references are in the style of PLOS Medicine (ICMJE/Vancouver style). The reference style guide can be found here: http://journals.plos.org/plosmedicine/s/submission-guidelines#loc-references. You can also find a downloadable EndNote file in these guidelines to assist you. Please do a manual check as well, as the EndNote file does not catch everything. Please note that the references will be typeset as they appear so please ensure the format is consistent. Checked.

2. Please put all reference citations in square brackets separated by either commas or dashes with no spaces, e.g. [1,2,3] or [1-3]. Amended.

3. Please make sure all references are cited in ascending numerical order in the text. Correct.

Comments from Reviewers:

Reviewer #1: The authors have addressed the majority of my comments on the initial version. I still have a few comments to the revision now presented:

1. The manuscript is still very extensive and not just in terms of word count. There are data

on AF prevalence and screening yield, comparison between diagnostic modalities, qualitative data and finally data on cost effectiveness.

2. I still find it difficult to follow all the figures presented in "AF prevalence". A flowchart would be of great help. We have constructed a flow chart (Fig 3), which we hope makes this section easier to follow.

3. The ECG findings and diagnosis presented in table 4 are mostly benign findings that will not have any relevance for asymptomatic patients, i.e. ectopics and most conduction abnormalities. I suggest toning down this part of the findings. A toned-down version of these findings has been included in the flow chart (Fig 3).

4. In "Future direction", it is stated that "The present study has demonstrated that coupling an AF screening initiative with the influenza vaccination programme is effective." The effectiveness of a screening program is defined as its ability to reduce the morbidity and mortality associated to the screened condition, this is not demonstrated with this study and I suggest rephrasing. We have changed this wording to "feasible".

5. Conclusion is still too long should preferably not contain references, rather conclusions based on data from the present study.

Agreed, we have removed this sentence and the reference from the conclusion.

6. The low performance of pulse palpation as a screening tool should be emphasized, and the superiority of the digital device over pulse palpation is one of the major results.

We have spent quite a bit of time talking about this in the discussion (line 551 to 565) and have it as a key point in the conclusion (line 606 to 608). However, we realise that this key point is missing from the abstract and have thus added to the Abstract Conclusion:

"Furthermore, diagnosis of AF by the clinical pharmacist using a $_{SL}$ECG was more sensitive and more specific than the use of pulse palpation alone."

We have also added another key reference (line 556) that has been published whilst this paper has been under review: Ghazal et al., 2020.

https://journals.plos.org/plosmedicine/article/file?id=10.1371/journal.pmed.1003063&type=printable

7. The suitability of a GP office as a screening central for AF to my opinion a theoretical construct. As partly mentioned in the introduction, the current staffing and workload in primary care is not compatible with additional tasks like AF screening, at least not beyond what is made by the tremendously committed investigators in local screening initiatives like in this one. There still very little evidence that AF screening could be done in the primary care setting

apart from delimited clinical trials, and to what extent can this study change that?

We agree that screening in a GP practice is not without its challenges, not just from a logistical aspect, but as you mention, it requires endorsement from the practice and its staff as well. We have added this as a limitation (Line 491):

"However, the space requirement, logistics and staff endorsement of such a screening strategy in some GP practices, may be prohibitive."

We have also emphasised this point better in the future direction section (Line 613 to 615):

"However, key questions remain relating to whether this model could be upscaled and delivered by pharmacists or other HCPs, in all GP practices and without the 'insurance' of a cardiologist screening all ECGs."

Reviewer #2: The Authors have provided a satisfactory response and the manuscript has been revised accordingly. I would recommend that the manuscript is accepted for publication. Thank you.

Reviewer #3: This revision is very responsive to my previous comments. Only a few thoughts remain:

1. Follow-up from previous review: I recommend replacing "non-parametric" variable with "continuous variable". The variable itself cannot be parametric (parametric assumptions are imposed by the analyst). This has been amended accordingly.

2. I still think the paper could be strengthened by discussion of implementation science type questions. While I agree with the authors that additional analyses are unnecessary, the discussion would be strengthened by considering points like "is the time required to train pharmacists on the methods needed worth the improvements noted" and whether the authors expect accuracy to decline as the intervention was scaled up (and moreover, what could be done to prevent this). These questions are important if the work is expected to inform future adoptions of this intervention in other settings. It might be that the authors believe that this approach can be scaled up without loss of accuracy due to important features of the tool, which would be important to note.

We agree that these are all important questions and would be points that we would try to address if we are given the opportunity to scale up this screening strategy in the future. We have tried to emphasise this better in the future direction section of the manuscript (Line 613 to 615):

"However, key questions remain relating to whether this model could be upscaled and delivered by pharmacists or other HCPs, in all GP practices and without the 'insurance' of a cardiologist screening all ECGs."

3. Based on the methods section, I expected Table 2 to compare sensitivity, specificity, positive predictive value, and false discovery rate. However, it is unclear what measure "accuracy" in Table 2 refers to? This should be clarified with a footnote or renamed.

In order to clarifiy this definition, we have amended the Methods section of the manuscript as follows:

"The overall diagnostic accuracy (correct classification rate) combined these two parameters as an assessment of the test's ability to detect both the proportions of true positives and true negatives (true positives and true negatives/total number of participants) [35]."

We have also added the term 'correct classification rate' to Table 2.

4. Page 9, line 192: the definitions for PPV and FDR should be clarified. PPV should be (# who both tested positive and were true positives) / (# who tested positive). Similarly the FDR should be (# who both tested positive and were true negatives)/(# who tested positive).

We have amended the 'Methods/ section of the manuscript as follows:

"The PPV and FDR were defined as probabilities that the test will identify those with positive diagnoses either correctly (PPV = number of participants who both tested positive and were true positives / total number who tested positive) or incorrectly (FDR = number of participants who both tested positive and were true negatives / total number who tested positive), respectively [34,36]."

Given that the FDR is simply 1-PPV, it may be more informative to report the PPV and the NPV (negative predictive value = (# true negatives who tested negative)/(# who tested negative). But I leave it up to the authors to determine the most useful metrics.

Whilst rarely reported [2,3], FDR helps appreciate the substantial rate of false positive misclassification by the index test, which may have both clinical and economic consequences, and may be as high as 86% for a highly accurate test displaying a 95% specificity for a disease of 1% prevalence [4]. This high rate of false discovery may not necessarily be obvious to an average reader from the value of PPV which focuses on the proportion of true positives. Considering the high false positive rates observed during our study, particularly with pulse palpation, we aimed to provide the values of FDR alongside the PPV to illustrate the impact these false positive discoveries may have. Regarding the NPV, this parameter focuses on the correct classification of those with disease negative diagnoses. It is therefore, highly dependent on the prevalence of the disease, and for low-prevalence conditions, such as AF, often approaches 100% [5-7]. As such, the added value of NPV to measures of sensitivity and specificity may be limited and subsequently we decided not to report them in this manuscript.
